# Genetic Mapping of a Major Resistance Gene to Pea Aphid (*Acyrthosipon pisum*) in the Model Legume *Medicago truncatula*

**DOI:** 10.3390/ijms17081224

**Published:** 2016-07-29

**Authors:** Lars G. Kamphuis, Su-Min Guo, Ling-Ling Gao, Karam B. Singh

**Affiliations:** 1 Commenwealth Scientific and Industrial Research Organisation, Agriculture and Food, 147 Underwood Avenue, Floreat, WA 6014, Australia; sg877@cornell.edu (S.-M.G.); lingling.gao@csiro.au (L.-L.G.); karam.singh@csiro.au (K.B.S.); 2 University of Western Australia Insititute of Agriculture, 35 Stirling Highway, Crawley, WA 6009, Australia; 3 Boyce Thompson Institute for Plant Research, Ithaca, NY 14853, USA

**Keywords:** barrel medic, legumes, insect resistance, resistance gene

## Abstract

Resistance to the Australian pea aphid (PA; *Acyrthosiphon pisum*) biotype in cultivar Jester of the model legume *Medicago truncatula* is mediated by a single dominant gene and is phloem-mediated. The genetic map position for this resistance gene, APR (*Acyrthosiphon pisum* resistance), is provided and shows that APR maps 39 centiMorgans (cM) distal of the *A. kondoi* resistance (AKR) locus, which mediates resistance to a closely related species of the same genus bluegreen aphid (*A. kondoi*). The APR region on chromosome 3 is dense in classical nucleotide binding site leucine-rich repeats (NLRs) and overlaps with the region harbouring the RAP1 gene which confers resistance to a European PA biotype in the accession Jemalong A17. Further screening of a core collection of *M. truncatula* accessions identified seven lines with strong resistance to PA. Allelism experiments showed that the single dominant resistance to PA in *M. truncatula* accessions SA10481 and SA1516 are allelic to SA10733, the donor of the APR locus in cultivar Jester. While it remains unclear whether there are multiple PA resistance genes in an R-gene cluster or the resistance loci identified in the other *M. truncatula* accessions are allelic to APR, the introgression of APR into current *M. truncatula* cultivars will provide more durable resistance to PA.

## 1. Introduction

Sap-sucking insects such as aphids, psyllids, scales and whiteflies cause significant damage in agricultural crops throughout the world. Damage is caused by direct feeding from the phloem sap as well as vectoring viruses, with aphids transmitting over 50% of all plant viruses [1]. Sap-sucking insects have a close association with their host and feed from a single cell type, the phloem sieve element. Sap-sucking insects have developed the ability to disguise their presence and/or suppress plant defences, ultimately leading to the establishment of a successful feeding site [2,3]. In recent years an increased research focus on studying plant—sap-sucking insect interactions has occurred, resulting in the identification of several sap-sucking insect resistance loci [4,5] and an improved understanding of the molecular mechanisms of basal defense as well as gene mediated resistance to sap-sucking insects is emerging [5].

The evolutionary origins of recognition of attackers of plants mainly stems from studies involving plant pathogens rather than insects and is better known as the plants innate immune system [6]. Recognition of an attacker often occurs through resistance (*R*) gene products which recognize specific attacker-derived product(s) and upon recognition mount a defence response. While these *R*-genes mediate resistance to a variety of different pathogens and pests, their architecture is highly similar and includes one of the following conserved motifs: Nucleotide binding site, leucine-rich repeat (NLRs) or serine/threonine protein kinase domains. This would imply that basic modes of recognition and subsequent signalling pathways that trigger the defence response have been retained through plant evolution and diversification [7,8].

An important advance in understanding *R*-gene mediated resistance to sap-sucking insects came from the discovery of the major dominant resistance gene *Mi1.2*, which confers resistance to three sap-sucking insects, being potato aphid (*Macrosiphum euphorbiae*), whiteflies (*Bemisia tabaci*) biotypes B and Q and psyllids (*Bactericerca cockerelli*) as well as three species of root-knot nematodes (*Meloidogyne* spp.) [9,10,11]. The second major *R*-gene identified and cloned was the *Vat* gene conferring resistance to cotton-melon aphid (*Aphis gossypii*) [12]. *Mi1.2* and *Vat* belong to the largest class of *R*-genes encoding proteins with NLR motifs of the subclass with coiled-coiled (CC) motifs. The silencing of the Resistance Gene Candidate 2 (RGC2) cluster of NLR encoding genes in lettuce (*Lactuca sativa*) led to the loss of resistance to the lettuce root aphid (*Phemphigus bursarius*) [13]. In the model legume *Medicago truncatula*, single dominant resistance genes to other aphid species including bluegreen aphid (BGA; *Acyrthosiphon kondoi*), spotted alfalfa aphid (*Therioaphis trifolii*) and pea aphid (PA; *Acyrthosiphon pisum*) map to regions dense in these NLR encoding genes [14,15,16,17]. For both *Mi1.2* and *Vat* as well as the single dominant resistance genes identified in *M. truncatula* resistance to aphids is exerted in the phloem, which shows that plants are able to utilize their innate immune systems to defend against parasitism of the phloem.

Over the last decade *M. truncatula* has emerged as an excellent model plant to study plant insect interactions [5,18], with major dominant resistance genes identified to bluegreen aphid [14], spotted alfalfa aphid [15] and pea aphid [17,19]. Furthermore, quantitative trait loci (QTLs) controlling different aspects of aphid resistance including antibiosis, antixenosis and tolerance to BGA, PA, spotted alfalfa aphid and cowpea aphid have been identified [20,21,22]. Resistance to BGA, PA and spotted alfalfa aphid has been introgressed into the *M. truncatula* variety Jemalong (A17) through recurrent backcrosses to create a new aphid-resistant cultivar Jester [19,23]. Resistance to these three aphid species in Jester has been dissected over the last decade and it was shown that in all cases it involves antibiosis and antixenosis, with resistance exerted at the phloem [14,15,24].

Resistance in *M. truncatula* to PA was of particular interest as PA has been chosen by the international aphid genome consortium (IAGC) as the model aphid and there is a reference genome sequence [25] and other genomic resources available [26] as well as a number of distinct PA biotypes [27]. In the case of the *Medicago*-PA interaction in Jester, it was unclear whether resistance to BGA and PA was conferred by the same single dominant resistance gene, *AKR* (*Acyrthosiphon kondoi* resistance). In 2009, Guo et al. demonstrated that resistance to the Australian PA biotype was introgressed into the Jester background from a different donor than the resistance to BGA, thus there were two distinct resistance genes for the Australian PA biotype and BGA, where the resistance locus to the Australian PA biotype was termed *APR* for *Acyrthosiphon pisum* resistance [19]. In *M. truncatula* resistance to an European pea aphid biotype (PS01) is distinct from resistance to the Australian biotype. Resistance to the European biotype was identified in *M. truncatula* accession A17 which is moderately resistant to the Australian biotype [17]. Like *APR* mediated resistance *RAP1* resistance is also exerted through the phloem. The genetic map position of *RAP1* is on linkage group 3 in a region harbouring both serine-threonine kinase and NLR proteins. *RAP1* mediated resistance causes 100% mortality to the European clone PS01 and is therefore different from *APR* mediated resistance since the antibiotic effect of *APR* on the Australian PA biotype shows no mortality, but rather a reduced reproductive rate [17,24].

Here we present a genetic map position for the *APR* locus and demonstrate that *APR* and *RAP1* map to the same region on chromosome 3. We also report on a screen of additional *M. truncatula* germplasm for PA resistance and elaborate on the hypotheses that *APR* and *RAP1* are two distinct genes tightly linked to one another in an *R*-gene cluster, or are alternative alleles of the same locus.

## 2. Results

### 2.1. Resistance to Pea Aphid in the Cultivar Jester Is Controlled by a Single Dominant Gene

**Figure 1:**
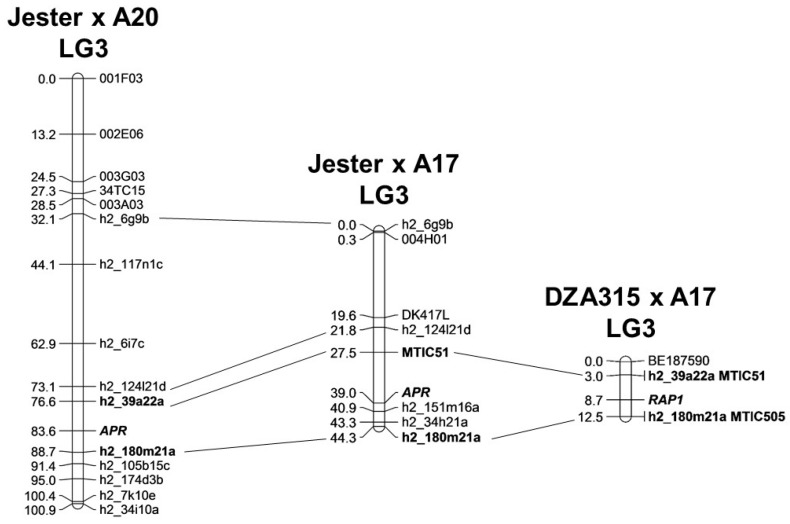
Genetic map position of the *APR* (*Acyrthosiphon pisum* resistance) locus conferring resistance to the Australian pea aphid biotype, covers the same region of interest as the region of interest for *RAP1* conferring resistance to a European PA biotype in the genetic background A17.

Previous mapping data suggested that PA resistance in Jester was linked to that of bluegreen aphid resistance mediated by the *AKR* locus on chromosome 3 [24]. To identify the genetic location of the *APR* locus, two genetic mapping populations were developed between Jester and A20, a wide cross as well as Jester and A17, a narrow cross. Molecular markers developed by the *M. truncatula* community [28,29,30], were screened for polymorphisms between the parents for each population (Table S1). A total of 129 F_2_ individuals were genotyped with 15 molecular markers polymorphic between Jester and A20. This resulted in the construction of a genetic linkage map for chromosome 3 spanning 100.9 centiMorgans (cM) with an average interval size of 7.2 cM. Seed was collected for these 129 individuals and their F_3_ offspring (*n* = 12 per F_3_ family) was infested with PA to determine their PA resistance response and thus the F_2_ alleles for the *APR* locus. This determined that the PA resistance locus *APR* is located between markers h2_39a22a and h2_180m21a spanning a 12.1 cM interval (Figure 1).

Jester and A17 are 89% identical in their genome organisation [19] with Jester mainly having a large insertion from different donors on chromosome 3. Therefore, the chance to identify recombinants in the *APR* region of interest from a cross derived between Jester and A17 is higher than that from a cross derived between Jester and A20; thus, 384 F_2_ individuals of the narrow cross derived between Jester and A17 were genotyped with eight polymorphic markers near the region of *APR* to identify individuals with recombination events around the *APR* locus. This identified a total of 26 individuals with recombination events in the *APR* region of interest and their F_3_ progeny (*n* = 12 per F_3_ family) were infested with PA to determine their resistance status. As shown in Figure 1 the region of interest for the *APR* locus in the Jester × A17 cross spans 13.4 cM between markers MTIC51 and h2_151m16a. This region spans a physical distance of 3972.4 Kb in the *M. truncatula* v4.0 genome assembly of accession A17, which harbours a cluster of classical nucleotide-binding site leucine-rich repeats (NLR) resistance genes, including the *RAP1* resistance gene to the European PA clone LS01 [17], but not the region where the bluegreen aphid resistance gene *AKR* has been mapped [14].

### 2.2. Screening of M. truncatula Accessions for Additional Sources of PA Resistance

With both *APR* and *RAP1* located in an NLR cluster on chromosome 3, we wanted to determine whether additional major PA resistance genes to the Australian PA biotype exist besides *APR* and perhaps with a more striking lethal resistance as conferred by *RAP1* to the European PA biotype LS01. Therefore, additional lines of *M. truncatula* were screened for aphid performance and plant damage. Thirty-five accessions of the South Australian Research and Development Institute (SARDI) *M. truncatula* core collection, which represent the major clades in the phylogenetic tree of the SARDI core accessions [31] were selected to evaluate PA resistance performance. These included accessions A20, Cyprus and Borung, previously identified as being highly susceptible to PA, A17 which is moderately resistant, as well as Jester and Caliph which are highly resistant to PA [32]. Plant damage and aphid populations were monitored over a 28-day period. One of the typical aphid infestation phenotypes in *M. truncatula* following infestation with PA is necrotic flecks on local leaves [17,24]; however this was only observed in *M. truncatula* accessions Jester and A17. No lethal resistance to PA was observed and all accessions showed varying degrees of stunting and wilting, with damage symptoms appearing as yellowing patches or leaf chlorosis surrounding the aphid infestation sites within 9 days after infestation. Nine accessions including two resistant controls (Jester and Caliph) were resistant and survived PA infestation after 28 days post infestation (dpi) and went on to flower and set seed, with the exception of one individual of accession SA27063 (Table 1). The remaining 26 accessions succumbed to the PA infestation, with 15 accessions including susceptible controls (Borung and A20) with higher plant damage scores than the moderately resistant accession A17 (Table 1).

In a subsequent experiment the nine resistant accessions and five highly susceptible accessions from the initial screen were infested to confirm their resistance response to PA infestation with A17 included as a moderately resistant control. Starting with the initial two adult apterous aphids, PA colony density on all susceptible accessions peaked around 12 dpi; thereafter, the plants succumbing to PA infestation by 15 dpi. PA population density on A17 plants, the moderately resistant accession, reached the peak around 15 dpi (Table S2), whereas aphid populations were the largest at 21 dpi on the resistant accessions and declined thereafter at 24 dpi (Table S2). Plant damage on resistant accessions SA1516, SA28645, SA10481, SA10733, Jester and SA11753 remained stable from 21 dpi onwards with an average score of 3.4 (Table S3).

**Figure 2:**
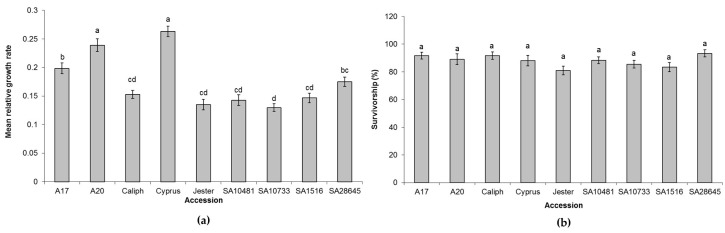
(**a**) Mean relative growth rate (MRGR) of pea aphid nymphs on nine *Medicago truncatula* accessions over four days. Values are mean and standard error of ten replicates. Accessions that do not share the same letters indicate significant differences in pea aphid MRGR from the other accessions by Tukey Kramer HSD test (*p* < 0.05); (**b**) Survivorship of pea aphid nymphs on nine *M. truncatula* accessions over four days. No significant differences were observed in survivorship by Tukey Kramer HSD test (*p* < 0.05).

There were some notable differences in the population sizes of PA on the different resistant accessions with a notably lower population density on SA1516 and SA10481 compared to Jester. In a follow-up short-term infestation experiment the performance of PA nymphs over a four-day period was observed, and this reflected the plant damage and aphid densities seen in the long term experiments (Figure 2). The PA nymph population had a significantly lower mean relative growth rate (MRGR) on Jester, SA10733, SA1516 and SA10481 compared to the moderately resistant A17, which, in turn, had a significantly lower MRGR compared to the highly susceptible accessions A20 and Cyprus (Figure 2a) (Tukey Kramer HSD test; *p* < 0.05). No significant differences between the accessions were found for the survivorship of PA nymphs over this four-day period (Figure 2b) (Tukey Kramer HSD test; *p* < 0.05).

### 2.3. Resistance in M. truncatula Accessions SA10733 and SA10481 Is Controlled by Single Dominant Gene

SA1516 and SA10481 had the lowest average plant damage scores, albeit similar resistance phenotype to Jester and SA10733, the donor of *APR* in cultivar Jester. Moreover, notably lower PA population densities on accessions SA1516 and SA10481 were observed in the long-term experiments. Therefore F_2_ populations were generated between the resistant accessions SA10733 and SA10481 and the highly susceptible accession A20 to determine the genetic control underlying the PA resistance in these accessions. Phenotyping of 264 and 355 F_2_ individuals of the SA10733 × A20 and SA10481 × A20 showed a Mendelian segregation ratio of 3:1 for PA resistance in both populations (Table 2).

To determine whether the single dominant resistance in SA10481 was allelic to that of SA10733 and/or SA1516, crosses were generated and F_2_ individuals for three crosses evaluated for their resistance to PA. As shown in Table 3 no susceptible individuals were identified, for any of the 535 individuals assayed, whereas the susceptible controls and moderately resistant controls behaved as seen in previous experiments. Thus the single dominant resistance in SA1516 and SA10481 and SA10733 are either alleles of the same gene (e.g., *APR*) or genes in a tightly linked resistance gene cluster.

## 3. Discussion

Previously, we have characterised PA resistance in the *M. truncatula* cultivar Jester, which also harbours resistance to bluegreen aphid [24]. The biology of the resistance to both aphid species in this cultivar shared similarities with resistances occurring at the phloem level and requires an intact plant and involves a combination of antibiosis, antixenosis and plant tolerance [14,24]. However, the donor for bluegreen aphid resistance (accession SA1499) was a different donor than that of PA resistance (accession SA10733), thus resistance to both aphids are controlled by distinct single dominant resistance genes with the PA resistance locus tentatively named *APR* [19]. Here we demonstrated that resistance to PA mapped 39 cM distal of the flanking markers for the bluegreen aphid resistance locus *AKR* (h2_6g9b and 004H01) on chromosome 3 in a region rich in classical NLR type of resistance gene (Figure 1). Moreover, the region that contains *APR* in the genetic background of Jester spans the same region as the region harbouring *RAP1* to the European PA biotype LS01 in the genetic background of A17 [17]. This could mean that *APR* and *RAP1* are either two different alleles of the same orthologous gene, or, alternatively, two different genes in a NLR cluster of resistance genes. Further fine-mapping will be achieved in future work by generating re-sequencing data for cultivar Jester to identify single nucleotide polymorphisms (SNPs) or insertions/deletions (indels) in the *APR* region with the 26 recombinant F_3_ families. This would narrow-down the region of interest further and allow a map-based cloning approach for the *APR* locus. Similarly, the use of the Medicago HapMap resources [33] that contains re-sequencing data for DZA315 would allow the identification of SNPs and indels to generate novel markers for further fine-mapping of the *RAP1* locus.

Screening of diverse *M. truncatula* accessions with eight different European biotypes has previously been conducted by Kanvil and colleagues [27] and showed a range of differences in performance of the different biotypes across 23 *M. truncatula* accessions. They demonstrated that aphid virulence and host resistance were strongly dependent on the genotype of both the aphid and the host where diverse host-specific PA performance and biotype specific resistance in *M. truncatula* were observed. In Australia, there is currently only one biotype present and in contrast to the study by Kanvil et al. [27], no lethal resistance to the Australian biotype was identified in *M. truncatula* germplasm. Despite this result, seven new accessions were identified as being resistant to PA at a similar level to SA10733 and Jester both harbouring the *APR* gene, with notably lower PA population densities on accessions SA1516 and SA10481 compared to current cultivar Jester (Figure 2, Table 1). To determine the genetic control of PA resistance in the resistant accessions, crosses were generated to the susceptible A20 and phenotyping of the F_2_ populations showed that resistance segregated in a Mendelian fashion for a single dominant gene (Table 2), raising the question whether the resistance identified in these accessions were allelic to *APR*, a gene somewhat linked to *APR* or an unlinked gene. Out of the 494 F_2_ individuals phenotyped none of them showed susceptibility, which suggests that the single dominant resistance in SA1516, SA10481 and SA10733 are either alleles of the same gene (e.g., *APR*) or genes in a tightly linked resistance gene cluster. The latter could be a valid hypothesis as the *RAP1* gene is also located in the same region on chromosome 3, and this region contains a suite of NLR resistance genes. The *RAP1* gene in *M. truncatula* provides race-specific resistance to pea aphid biotype PS01 but not to biotype LL01 [17]. Furthermore, it has been shown that different PA biotypes (both sexual and asexual clones) differ in their performance on a range of *M. truncatula* accessions, including Jester and A17 plants [27]. Another PA biotype, N116, was virulent on RAP1 genotypes like biotype LL01 as well as on a wide range of other cultivars and wild *M. truncatula* genotypes [27]. On the contrary, PS01 was avirulent on most of the *M. truncatula* accessions. The divergent performance of these PA biotypes allowed the determination of inheritance of aphid virulence, and it was demonstrated through a series of F_1_ progenies of clones N116 and PS01 that the RAP1 mediated resistance can be overcome by progeny from either selfing or reciprocal crosses [34]. This suggests that the annual sexual cycle in aphids can lead to the generation of novel genotypes, which might have increased or decreased virulence. In turn, *M. truncatula* has to adapt and develop new forms of resistance to PA. In other plant species, this adaptation to other forms of virulent pathogens/pests occurs according to the birth and death model of *R* genes where *R*-genes duplicate and diversify in gene clusters [35]. Further fine-mapping of the identified PA resistance loci would shed more light on whether this has occurred in *M. truncatula* in response to different PA biotypes.

**Figure 3:**
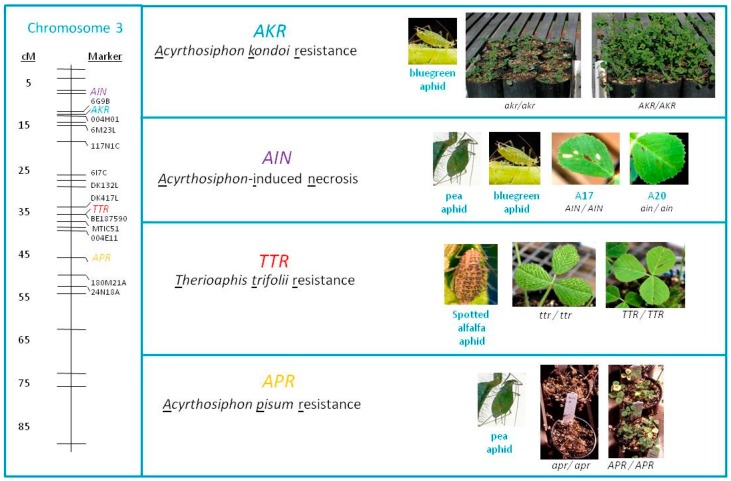
Overview of the major resistance genes identified in *M. truncatula* cv. Jester to three different aphid species.

The identification of the *APR* resistance gene in *M. truncatula* cv. Jester is the fourth major aphid resistance gene in this genetic background (Figure 3), which also harbours resistance to bluegreen aphid conferred by genes *AKR* [14] and *AIN* [16] and spotted alfalfa aphid conferred by *TTR* [15]. Breeders introgressed resistance to bluegreen aphid and spotted alfalfa aphid into the genetic background of Jemalong A17 from various resistance sources [19,23]. Since the *APR* locus is located 10.5 cM distal of the flanking marker for *TTR* in the Jester x A20 population and thus somewhat linked to *TTR*, they coincidentally introduced resistance to PA as well (Figure 3). The wealth of *M. truncatula* genomic resources including a reference genome sequence for Jemalong A17 [36,37] and a genome sequence for the model aphid PA [25] makes the *M. truncatula-*PA system a great one to study plant-insect interactions and *R* gene specificity and evolution. Similarly, PA genomic datasets such as numerous Expressed Sequence Tags (EST) and transcriptome resources [38] and RNA interference methods to silence aphid genes [39,40] would complement the plant based studies and allow the identification of aphid effectors recognised by the resistance genes. The use of these resources and in addition to the advances in sequencing technologies and Clustered regularly interspaced short palindromic repeats (CRISPR)/Cas9 should allow the development of new ways to identify essential PA genes to establish a feeding site and/or effectors recognized by the resistance locus and might lead to effective durable resistance to aphids.

## 4. Materials and Methods

### 4.1. Plants and Aphids

Three genotypes of *M. truncatula* were mainly used being: Jester, A17 and A20. Genetic F_2;3_ mapping populations derived from crosses derived between both Jester and A20, and Jester and A17, were generated using a crossing procedure described by Thoquet et al [41] and used in this study for the genetic mapping and phenotyping for PA resistance. The *M. truncatula* core collection accessions were acquired from the South Australian Research and Development Institute (SARDI, Urrbrae, Australia). Accessions DZA315 and DZA045 were obtained from the Institut National de la Recherche Agronomique (INRA), Montpellier, France. Seeds were germinated and plants grown as described by Klingler et al. [16]. The aphid species used was PA collected in Western Australia and were reared on faba bean (*Vicia faba*), as described by Gao et al. [32].

### 4.2. Plant Damage and PA Performance Tests

To assess the performance of PA and plant feeding damage, two-week-old seedlings of *M. truncatula* lines A17, A20 and Jester as well as 129 F_3_ families (*n* = 12 per F_3_ family) of the Jester × A20 population and 26 F_3_ families (*n* = 12 per F_3_ family) of the Jester × A17 population were grown in separate 0.9 L pots and were infested with two apterous adult aphids. Similarly, the 35 accessions (Table 1) were screened for PA resistance in a glasshouse when two-week-old and infested with two apterous adult aphids. The screening of the 35 accessions was arranged in a randomized complete block design with three replicates per accession infested for 28 days.

In all phenotyping experiments the aphids were allowed to develop, reproduce, and move freely among plants. Aphid population build-up and feeding damage on plants were assessed at a three-day interval from the third day up to 28 days post infestation using a scale from 1–5 and 0–5, respectively as described previously [20].

### 4.3. Aphid Performance on Caged Leaves

The survival and growth rate of PA were measured after four days on individual plants of each *M. truncatula* accession with ten replicates for each accession and the mean relative growth rate (MRGR) calculated as described by Gao et al. [32]. The proportion of aphids that survived and MRGR were compared using the Tukey-Kramer Honestly Significant Difference test with the JMP-IN 5.1 software (SAS Institute, Cary, NC, USA).

### 4.4. Genetic Mapping of PA Resistance in the Various Mapping Populations

Genetic maps for the Jester × A20 and Jester × A17 mapping populations were generated using both microsatellite and gene-based markers generated by the *Medicago* research community. Previously we established linkage association with markers on linkage group 3 [24] and therefore markers were initially selected to be evenly distributed over linkage group 3 and were obtained from several published sources [28,29,30]. A total of 26 markers were characterised for the Jester × A20 (*n* = 129) and for Jester × A17 (*n* = 384) populations with the polymorphic markers for the respective populations listed in Table S1.

Linkage group 3 was constructed for both mapping populations using a set of 15 and 8 markers for the Jester × A20 and the Jester × A17 population respectively, using Multipoint v1.2 (Institute of Evolution, Haifa University, Haifa, Israel) as described by Kamphuis et al. [42].

### 4.5. Allelism Tests

Pairwise crosses were made among SA10733, SA1516 and SA10481 to test the allelic status of the PA resistance in SA1516 and SA10481 as in Table 3. The seedlings of F_2_ from each cross with at least eight replicates of their respective parental genotypes and A20 were tested for PA resistance. Each three-to-four-week-old seedling was infested with two apterous adult PAs for 28 days. During this period, aphids were allowed to develop, reproduce and move freely. Aphid resistance were scored as either resistant or susceptible at 28 dpi. Susceptible plants die before 20 dpi and with overwhelming aphids around 12 dpi and then totally migrate to the other plants due to the death of the host plant; resistant plants are still surviving at 28 dpi and reasonably healthy. The appearance of parental lines and A20 was used as controls.

## Figures and Tables

**Table 1:** Evaluation of 35 *Medicago truncatula* accessions from the South Australian Research and Development Institute (SARDI) core collection for resistance to an Australian biotype of pea aphid. Each value represents the mean and standard error (SE) of three biological replicates. For the aphid population build-up, the rating scale was as described by Gao et al. [32].

Accession	Aphid Score 9 dpi (SE)	Plant Score 15 dpi (SE)	Plant Score 21 dpi (SE)	Plant Survivorship 28 dpi	Comment
SA11753	2.5(0.3)	1.6(0.7)	3.0(0.0)	3/3	Resistant
SA28645	2.5(0.9)	2.2(0.3)	3.0(0.1)	3/3	Resistant
SA3047	1.8(0.6)	2.5(0.3)	3.1(0.1)	3/3	Resistant
SA10481	2.8(0.4)	2.3(0.3)	3.3(0.2)	3/3	Resistant
SA1516	1.7(0.2)	2.5(0.3)	3.6(0.5)	3/3	Resistant
SA27192	1.7(0.2)	1.3(0.1)	3.6(0.6)	3/3	Resistant
SA27063	2.3(0.3)	3.5(0.3)	3.6(0.4)	2/3	Resistant
Caliph	1.8(0.3)	2.4(0.3)	3.8(0.4)	3/3	Resistant (control)
Jester	1.5(0.3)	2.7(0.2)	3.9(0.2)	3/3	Resistant (control)
SA25654	2.3(0.3)	2.0(0.6)	3.3(0.3)	0/3	Moderately susceptible
SA18395	1.2(0.2)	2.1(0.6)	3.5(0.0)	0/3	Moderately susceptible
SA8604	2.3(0.2)	2.0(0.6)	4.0(0.4)	0/3	Moderately susceptible
SA9062	2.3(0.3)	2.3(0.3)	4.0(0.4)	0/3	Moderately susceptible
SA30199	2.2(0.4)	2.3(0.2)	4.1(0.2)	0/3	Moderately susceptible
SA3569	2.2(0.4)	2.2(0.3)	4.3(0.3)	0/3	Moderately susceptible
SA10419	2.7(0.7)	4.3(0.6)	4.4(0.6)	0/3	Moderately susceptible
DZA315	2.8(0.6)	4.0(0.1)	4.6(0.1)	0/3	Moderately susceptible
SA17590	2.8(0.7)	3.0(0.6)	4.6(0.2)	0/3	Moderately susceptible
A17	2.7(0.2)	3.0(0.5)	4.7(0.1)	0/3	Moderately susceptible (control)
SA3919	1.7(0.4)	2.3(0.6)	4.7(0.2)	0/3	Susceptible
SA24968	2.2(0.4)	3.3(0.7)	4.8(0.1)	0/3	Susceptible
SA3054	2.8(0.4)	2.9(0.9)	4.8(0.1)	0/3	Susceptible
SA8618	3.2(0.2)	3.3(0.3)	4.8(0.3)	0/3	Susceptible
SA11734	2.5(0.3)	4.1(0.5)	4.9(0.1)	0/3	Susceptible
SA9357	3.8(0.2)	4.3(0.2)	4.9(0.1)	0/3	Susceptible
SA22323	3.3(0.3)	4.3(0.3)	5.0(0.0)	0/3	Susceptible
SA7749	3.2(0.3)	4.6(0.1)	5.0(0.0)	0/3	Susceptible
SA9710	2.5(0.3)	4.5(0.3)	5.0(0.0)	0/3	Susceptible
SA9712	2.7(0.2)	4.4(0.2)	5.0(0.0)	0/3	Susceptible
Cyprus	3.0(0.6)	4.5(0.3)	5.0(0.0)	0/3	Susceptible (control)
Borung	3.3(0.2)	4.9(0.1)	5.0(0.0)	0/3	Susceptible (control)
A20	3.7(0.2)	4.8(0.1)	5.0(0.0)	0/3	Susceptible (control)
SA1499	3.3(0.2)	4.4(0.2)	5.0(0.0)	0/3	Susceptible
DZA045	3.0(0.5)	4.8(0.1)	5.0(0.0)	0/3	Susceptible
SA1489	3.7(0.2)	4.9(0.1)	5.0(0.0)	0/3	Susceptible

**Table 2:** Segregation of resistance to pea aphid in resistant *M. truncatula* accession crossed with accession A20. Chi-square analysis for a single dominant Mendelian inheritance of resistance of the two F_2_ populations indicates single dominant, Mendelian inheritance of resistance to PA in both populations.

Population	Resistant: Susceptible			
	Observed	Expected	χ^2^	*p*
SA10733 × A20	200:64	198:66	0.081	0.776
SA10481 × A20	264:91	266:89	0.076	0.783

**Table 3:** Pairwise allelism test between resistant *M. truncatula* accessions. Chi-square analysis for two unlinked Mendelian dominant genes indicates the resistance genes are either allelic or tightly linked.

Population	Resistant: Susceptible			
	Observed	Expected	χ^2^	*p*
SA10733 × SA1516	144:0	135:9	9.6	0.0019
SA1516 × SA10481	100:0	93.75:6.25	6.667	0.010
SA10481 × SA10733	250:0	234.4:15.6	16.667	0.00005

## Supplementary Materials

Supplementary materials can be found at http://www.mdpi.com/1422-0067/17/8/1224/s1.
