# Supplementary material for: Genetic Mapping of a Major Resistance Gene to Pea Aphid (Acyrthosipon pisum) in the Model Legume Medicago truncatula
Source: Int J Mol Sci. 2016 Jul 29;17(8):1224. doi: 10.3390/ijms17081224 (PMC5000622; doi:10.3390/ijms17081224)
Supplement: Supplementary file 1 [file ijms-17-01224-s001.pdf]

Supplementary Materials: Genetic Mapping of a Major Resistance Gene to Pea Aphid (*Acyrthosipon pisum*) in the Model Legume *Medicago truncatula*

Lars G. Kamphuis, Su-Min Guo, Ling-Ling Gao and Karam B. Singh

Table S1. Overview of the molecular markers used for the genotyping of the Jester x A17 and Jester x A20 F_2 mapping populations.

Molecular Marker Name	Forward Primer	Reverse Primer	A17 Fragment Length (bp)	Jester Fragment Length (bp)	A20 Fragment Length (bp)
001F03	catgtaacgcttgaggctga	caaccctaaccctcaccaaa	242	240	221
002E06	ctgatcaattgaaacaaacg	ctctcaccctctcatttgaa	150	150	136
003G03	aagcaagcaatcatcattatcaaa	aacgatgtgttctatgcgga	280	280	255
34TC15	aaatgatccattgccaccat	tgtgaatggaacaacaaaaag	285	285	266
003A03	agggaccggagaatttacatc	tcatggtacaatacatggagcaa	363	364	312
h2_6g9b	cccgatcgcagacataaaat	aagccaaatccattgcttga	302	292	317
004H01	tcccaatttatgttgtcctc	ttgttatgaaggggaagaga	209	219	219
h2_117n1c	tgcacaactctaatgcaagc	atgaagtcttggcgaggaaa	274	275	285
h2_6i7c	gggaaaatagtttcggaggg	accgcataaccaaacacact	304	291	237
DK417L (BbvI)	actcgtcgcctaacaatatcaaccag	gaattccatatccaacaccttta gactta	410	180 + 230	180 + 230
h2_124l21d	tcacttctaggcggtgacag	cttggccctataaacaggtca	183	187	164
h2_39a22a	acctcaactccacacacaca	gcacctgggaagggtaaaag	260	229	236
MTIC51	agtatagtgatgaagtggtagtgaaca	acaaaaactctcccggcttt	151	138	139
h2_151m16a	tgtagtgcctagcatgtgttga	ggcacagctatgagatatataggg	231	202	202
h2_34h21a	aaaagcatgtgatgtgggtg	aggttgtgcattttgcagtg	133	138	138
h2_180m21a	ttctccccatttttgctcac	ccaaacgcaccactctctct	319	316	296
h2_105b15c	tgttgtgttagcctctctctctc	agcggtgtttttgtccaact	208	180	171
h2_174d3b	tgattgcagtctttctggga	gccgctaggctacttcacaa	178	178	155
h2_7k10e	taaggcaatgtcagtcgcaa	gcatgcttttatgccctctc	244	244	227
h2_34i10a	tgtggcaggtgaatcatattg	tgaacgcagtaacgtggaga	244	245	239

Table S2. Mean aphid population score for selected accessions over a time-course observation following PA infestation. Each value represents the mean and SE of five biological replicates.

Accession	3 dpi (SE)	6 dpi (SE)	9 dpi (SE)	12dpi (SE)	15 dpi (SE)	18 dpi (SE)	21 dpi (SE)	24 dpi (SE)
SA1516	1.10 (0.06)	1.50 (0.14)	1.55 (0.09)	2.00 (0.26)	3.45 (0.22)	3.70 (0.18)	4.20 (0.20)	3.55 (0.20)
SA10733	1.00 (0.00)	1.30 (0.05)	1.50 (0.00)	2.40 (0.29)	3.45 (0.23)	3.50 (0.21)	4.00 (0.00)	3.00 (0.00)
SA10481	1.10 (0.06)	1.60 (0.06)	1.95 (0.05)	2.40 (0.32)	3.70 (0.20)	3.75 (0.14)	4.00 (0.00)	3.25 (0.19)
Jester	1.00 (0.00)	1.55 (0.09)	1.60 (0.10)	3.25 (0.22)	3.65 (0.19)	3.55(0.20)	4.00 (0.00)	3.00 (0.00)
SA27063	1.05 (0.05)	1.50 (0.08)	1.85 (0.10)	3.30 (0.43)	3.75 (0.16)	4.35 (0.27)	4.40 (0.24)	2.80 (0.72)
SA27192	1.05 (0.05)	1.55 (0.24)	2.00 (0.18)	3.30 (0.45)	3.55 (0.20)	4.40 (0.24)	2.80 (1.16)	3.00 (1.22)
SA3047	1.05 (0.05)	1.55 (0.15)	1.95 (0.05)	3.35 (0.47)	3.90 (0.10)	4.00 (0.00)	4.20 (0.20)	2.00 (0.82)
SA28645	1.10 (0.06)	1.55 (0.05)	2.05 (0.18)	3.40 (0.20)	3.65 (0.38)	3.55 (0.18)	4.00 (0.00)	2.55 (0.64)
SA11753	1.05 (0.05)	1.95 (0.31)	2.15 (0.17)	3.50 (0.21)	3.70 (0.34)	3.50 (0.22)	3.70 (0.20)	2.60 (0.68)
A17	1.25 (0.14)	2.00 (0.00)	2.85 (0.13)	3.85 (0.06)	3.85 (0.10)	1.90 (0.78)	0.00 (0.00)	0.00 (0.00)
Cyprus	1.25 (0.08)	1.65 (0.06)	2.60 (0.23)	3.95 (0.32)	2.80 (1.16)	0.00 (0.00)	0.00 (0.00)	0.00 (0.00)
SA1499	1.25 (0.08)	1.85 (0.10)	3.45 (0.20)	3.80 (0.97)	1.00 (1.00)	0.00 (0.00)	0.00 (0.00)	0.00 (0.00)
Borung	1.30 (0.09)	1.80 (0.09)	2.70 (0.27)	4.40 (0.24)	0.00 (0.00)	0.00 (0.00)	0.00 (0.00)	0.00(0.00)
SA1489	1.25 (0.11)	1.75 (0.11)	3.20 (0.24)	4.80 (0.20)	0.00 (0.00)	0.00 (0.00)	0.00 (0.00)	0.00 (0.00)
DZA045	1.20 (0.05)	2.00 (0.27)	3.25 (0.27)	3.40 (0.87)	0.00 (0.00)	0.00 (0.00)	0.00 (0.00)	0.00 (0.00)

Table S3. Mean plant damage score for selected accessions over a time-course observation following PA infestation. Each value represents the mean and SE of five biological replicates.

Accession	3 dpi (SE)	6 dpi (SE)	9 dpi (SE)	12 dpi (SE)	15 dpi (SE)	18 dpi (SE)	21 dpi (SE)	24 dpi (SE)
SA1516	0.25 (0.11)	1.10 (0.15)	1.10 (0.06)	1.50 (0.18)	2.05 (0.20)	2.85 (0.17)	3.45 (0.05)	3.35 (0.06)
SA28645	0.25 (0.11)	0.70 (0.15)	0.90 (0.15)	1.50 (0.21)	2.45 (0.12)	3.00 (0.14)	3.35 (0.17)	3.35 (0.23)
SA10481	0.40 (0.10)	1.40 (0.10)	1.25 (0.08)	1.75 (0.08)	2.45 (0.18)	3.25 (0.18)	3.45 (0.05)	3.45 (0.12)
SA10733	0.10 (0.06)	0.95 (0.05)	1.20 (0.15)	1.85 (0.15)	2.20 (0.05)	3.05 (0.05)	3.45 (0.05)	3.45 (0.15)
Jester	0.85 (0.10)	1.70 (0.15)	1.80 (0.09)	2.00 (0.00)	2.55 (0.12)	3.45 (0.05)	3.60 (0.06)	3.50 (0.11)
SA11753	0.45 (0.18)	1.65 (0.19)	1.50 (0.39)	2.55 (0.17)	2.95 (0.09)	3.30 (0.15)	3.55 (0.12)	3.55 (0.18)
SA27063	0.55 (0.09)	1.60 (0.19)	1.70 (0.12)	2.45 (0.39)	2.40 (0.13)	3.25 (0.11)	3.60 (0.06)	4.05 (0.15)
SA3047	0.15 (0.06)	1.00 (0.08)	1.10 (0.06)	2.65 (0.46)	2.30 (0.12)	3.30 (0.09)	3.75 (0.08)	4.15 (0.15)
SA27192	0.35 (0.06)	1.20 (0.09)	1.15 (0.10)	2.10 (0.42)	1.95 (0.22)	3.10 (0.28)	4.10 (0.32)	4.30 (0.29)
A17	1.25 (0.11)	2.00 (0.00)	2.40 (0.06)	2.95 (0.05)	3.30 (0.15)	4.05 (0.09)	4.35 (0.10)	4.65 (0.13)
Cyprus	0.50 (0.18)	1.35 (0.19)	1.20 (0.09)	2.90 (0.64)	4.30 (0.22)	4.90 (0.06)	5.00 (0.00)	5.00 (0.00)
SA1499	0.95 (0.05)	1.85 (0.10)	2.10 (0.10)	3.75 (0.29)	4.60 (0.13)	4.95 (0.05)	5.00 (0.00)	5.00 (0.00)
Borung	0.25 (0.08)	1.10 (0.20)	1.70 (0.31)	3.70 (0.44)	4.95 (0.05)	5.00 (0.00)	5.00 (0.00)	5.00 (0.00)
SA1489	0.35 (0.10)	1.55 (0.05)	1.85 (0.23)	4.25 (0.21)	4.90 (0.06)	5.00 (0.00)	5.00 (0.00)	5.00 (0.00)
